# Frequency and Association between Adverse Childhood Experiences and Psychiatric Outcomes Among Treatment-seeking Patients with Severe Obesity: a Retrospective Observational Study

**DOI:** 10.1007/s11695-026-08883-z

**Published:** 2026-08-18

**Authors:** Mariane Carvalho Cremonesi, Leorides Severo Duarte-Guerra, Denis Pajecki, Marco Aurélio Santo, Francisco Lotufo-Neto, Yuan-Pang Wang

**Affiliations:** 1 Instituto & Departamento de Psiquiatria (LIM-23), Hospital das Clínicas, Faculdade de Medicina, Universidade de São Paulo, São Paulo, Brazil; 2 Departamento de Cirurgia Gástrica, Universidade de São Paulo, São Paulo, Brazil

**Keywords:** Adverse childhood experiences, Childhood maltreatment, Obesity, Bariatric surgery, Psychiatric disorders

## Abstract

**Introduction:**

Adverse childhood experiences (ACEs) are well-established risk factors for obesity and psychiatric disorders in adulthood. Despite growing evidence, the association of specific ACE categories with the mental health of patients with severe obesity remains poorly understood. This study aimed to evaluate the frequency of ACEs and the association of ACEs with mental conditions among patients who underwent metabolic bariatric surgery.

**Methods:**

A total of 141 were recruited from a specialized bariatric center. ACEs were assessed using the Childhood Experiences section of the Composite International Diagnostic Interview 3.0. Psychiatric disorders (current and lifetime) were evaluated using the Structured Clinical Interview for DSM-5.

**Results:**

The sample (83.0% women; mean age 52 years; mean BMI 34.2 kg/m²) showed that 91.5% of patients reported at least one ACE, with maltreatment being the most frequent (81.6%). Among the ACE categories, family maladjustment was associated with the highest number of psychiatric disorders, both in current and lifetime assessments. Specifically, it was significantly associated with bipolar disorder (current: OR = 2.9; lifetime: OR = 3.5), any substance use disorder (current: OR = 4.3; lifetime: OR = 5.3), and any anxiety disorder (current: OR = 2.9; lifetime: OR = 2.3). The number of ACEs was associated with current psychiatric disorders (*p* = 0.04), where each additional ACE increased the odds of current psychiatric disorders by 7% (OR = 1.1, 95% CI: 1.0–1.1).

**Conclusions:**

Patients with severe obesity presented a high frequency of ACEs, which was associated with a later risk of developing psychiatric disorders.

## Key Points

- 91.5% of patients who underwent metabolic bariatric surgery reported at least one ACE.
- Family maladjustment is associated with bipolar, anxiety and substance use disorders.
- Each additional childhood trauma was associated with 7% higher odds of current psychiatric disorders.

## Introduction

Adverse childhood experiences (ACEs) encompass traumatic events occurring before the age of 18, including abuse, neglect, and dysfunctional family environments [1, 2]. Extensive research has shown that ACEs have a profound and lasting impact on health, even in individuals who do not fully meet the criteria for post-traumatic stress disorder (PTSD) [3, 4]. Exposure to early life adversities is associated with an increased risk of chronic diseases, particularly psychiatric disorders, and premature mortality [1, 5]. The underlying biological mechanism involves “toxic stress”, the enduring activation of the stress response, which dysregulates neurological, endocrine, immune, and metabolic systems [6, 7]. Within this context, individuals exposed to early-life adversity exhibit a 42% higher probability of developing obesity in adulthood, reflecting the long-term association between chronic stress and metabolic functioning [8].

Obesity and psychiatric disorders frequently co-occur. Previous studies indicated that individuals seeking metabolic bariatric surgery (MBS) frequently presented with a range of mental health conditions, most notably depressive and anxiety disorders, as well as binge eating disorder [9, 10]. These psychiatric comorbidities not only affect the quality of life but also intersected with body image concerns and psychosocial functioning [11, 12]. The co-occurrence of these conditions has been well established, growing clinical recognition of their relevance for bariatric patient care has led to an increasing number of publications examining the role of psychopathology among bariatric surgery candidates [8, 13, 14].

Individuals with a history of ACEs face elevated risks of developing psychiatric disorders, maladaptive coping strategies, and emotional regulation challenges. Unresolved trauma may be linked to weight regain and poor mental health outcomes following MBS [15, 16]. These factors substantially impact preoperative psychological readiness and postoperative adaptation [17, 18]. Although psychiatric disorders are common among individuals seeking MBS, data on prior ACE exposure in this population was often overlooked in clinical practice and insufficiently investigated [10, 17, 19]. Investigating specific types and cumulative associations of ACEs can offer insights into the interplay between early-life stress and long-term outcomes in these patients [17, 19]. Moreover, implementing trauma-informed care (TIC) framework can mitigate the risk of re-traumatization during the perioperative period and to foster a clinical environment that prioritizes long-term psychosocial healing and patient-centered stability [20].

Addressing this knowledge gap may contribute to improved clinical assessment and patient care. Identifying specific levels of early-life adversity linked to psychopathology may strengthen recommendations for ACE screening and clinical management within obesity management programs. This study aimed to evaluate the association between cumulative exposure to specific categories of ACEs and psychiatric disorders among patients who sought MBS.

## Methods

### Study Design and Sampling

This study was part of a larger cross-sectional project initiated in 2011, which with the aim of assessing the frequency of psychiatric disorders in patients with severe obesity who were candidates for MBS. At baseline, 500 patients on the waiting list of a specialized bariatric center were contacted, and 378 agreed to participate in the first phase of the study. In 2019, approximately eight years post-baseline, a follow-up assessment added the evaluation of ACEs. The current sample comprised 141 individuals with obesity who underwent MBS and completed clinical interviews in both study phases.

Individuals were excluded if they presented psychosis or intellectual disability, as such conditions often represent clinical contraindications for the surgical procedure. Other exclusion criteria included being under 18 years of age, illiteracy, or having mobility or geographical constraints.

The institutional ethics committee approved the study (CAPPesq #0228/11). All participants provided informed consent before participating in the study.

### Assessment Tools

#### Adverse Childhood Experiences (ACEs)

Adverse childhood experiences (ACEs) were assessed using the World Mental Health Survey version of the Composite International Diagnostic Interview (WMH-CIDI), which includes a structured section on childhood adversities experiences [21–25]. The 12 childhood adversity variables assessed by the WMH-CIDI were categorized for analysis into four conceptually distinct groups, following frameworks previously adopted in studies from the World Mental Health Survey Initiative [23, 24]. This classification distinguishes between: (1) interpersonal losses (e.g., caregiver death, parental divorce, or leaving home before age 18); (2) family maladjustment (caregiver mental illness, substance misuse, suicide attempt, criminality, and family violence); (3) mistreatment (sexual/physical abuse and neglect); and (4) life-threatening illness.

Parental maladjustment, including mental health disorders and substance misuse, was evaluated using the Family History Research Diagnostic Criteria Interview [25, 26]. This assessment covered specific psychiatric conditions such as depression, generalized anxiety disorder and panic disorder in either male or female caregiver. Family violence and instances of physical abuse were measured using a modified version of the Conflict Tactics Scale [27], capturing experiences such as being hit, slapped or having objects thrown by primary caregivers. Parental neglect was identified through questions adapted from child welfare research [28], addressing issues like lack of appropriate supervision, being assigned tasks unsuitable for the child’s age, and insufficient access to basic needs such as food, clothing, or medical care. Although the classification of interpersonal losses as core ACEs is not universally accepted across all conceptual frameworks, these variables were included in accordance with expanded international frameworks due to their documented impact on adult psychopathology [23, 24]. The module also includes an item assessing the presence of serious or life-threatening physical illnesses during childhood [29]. The WMH-CIDI was translated, cross-culturally adapted, and fully validated for the Portuguese-speaking population in Brazil (specifically utilized in large epidemiological study conducted in São Paulo (Brazil), São Paulo Megacity Mental Health Survey) [30, 31].

#### Structured Clinical Interview for DSM-5

Psychiatric disorders were assessed utilizing the Structured Clinical Interview for DSM-5 (SCID-5) [32], a standardized semi-structured interview for diagnosing psychiatric disorders listed in DSM-5. After screening for the presence of core psychopathology, the interviewer determined if the respondent meets the criteria for affective disorders (sections A, D), psychotic disorders (C, D), substance use disorders (SUD) (E), anxiety disorders (F), somatoform disorders (G), eating disorders (H), and adjustment disorders (I). The psychotic and somatoform sections were omitted to optimize administration time. Nine trained clinicians conducted interviews under continuous supervision. The study included an initial calibration phase for instrument familiarization, during which one professional conducted the interview while two others simultaneously observed the assessment. Each evaluator independently recorded whether the patient met the diagnostic criteria to ensure consistency across raters. Inter-rater reliability was measured using Fleiss’ Kappa coefficient, resulting in a value of *k* = 0.72, which was considered satisfactory. Throughout the data collection period, clinical interviews were conducted under the continuous supervision of a post-doctoral researcher and a senior supervising professor. The Brazilian Portuguese version of the SCID-5-CV demonstrated robust psychometric properties. The validation study showed high diagnostic sensitivity and specificity (both > 0.70) when compared to expert consensus diagnoses. The same study also reported excellent interrater reliability, with kappa coefficients exceeding 0.70 for most diagnostic categories [33, 34]. After the interview, the clinician scored the Global Assessment Functioning (GAF). This scale was used to quantify the impact of psychiatric symptoms on the patient’s overall psychological, social, and occupational functioning [35–37].

#### Statistical Analysis

A descriptive analysis was conducted to summarize the demographic variables such as sex, age, marital status, years of education, BMI, and ACEs. The ACEs were categorized into interpersonal losses, family maladjustment, maltreatment, and life-threatening illness in childhood. Proportion (%) and standard deviation (SD) were estimated for each variable.

Logistic regression models were used to determine whether ACE categories were associated with current and lifetime DSM-5 disorders and specific disorders such as bipolar, depressive, substance, anxiety, and eating disorders. Additionally, logistic regression assessed if the number of ACEs was associated with the presence of any current or lifetime psychiatric disorder. Associations between ACEs and psychiatric disorders were estimated using odds ratios (ORs) and 95% confidence intervals (CI). The logistic models were controlled by sex, age, GAF, BMI and time after MBS.

The data analysis was performed using R version 4.0.2. A significance level of 5% was adopted for all analyses with two-tailed tests.

## Results

Table 1 displays the demographic characteristics of the sample (*N* = 141). The majority were female (83.0%) and married (51.8%). Education level was predominantly between 9 and 12 years (38.3%). The mean age was 52.0 years (SD = 10.0), and the mean BMI was 34.2 kg/m² (SD = 5.6).

**Table 1:** Demographic characteristic of the sample (*N* = 141)

Characteristic	*n*	(%)
Sex		
Female	117	83.0
Male	24	17.0
Marital status		
Single	24	17.0
Married	73	51.8
Separated/divorced	28	19.9
Widowed	16	11.3
Education		
1–8 years	48	34.0
9–12 years	54	38.3
> 12 years	39	27.7
mean age, year (SD)	52 (10.0)	
mean BMI, kg/m^2^ (SD)	34.2 (5.6)	

Table 2 displays the distribution of ACE among adults with severe obesity. Within this sample, 129 individuals (91.5%) reported exposure to at least one ACE. All men reported experiencing at least one ACE, whereas the percentage for women was 89.7%. The most frequent categories were maltreatment (81.6%) and family maladjustment (72.9%). The category with the lowest frequency was life-threatening illness (11.5%).

**Table 2:** Frequency of adverse childhood experiences among adults who underwent bariatric surgery

Adverse Childhood Experiences	Female *n* (%)	Male *n* (%)	Total *n* (%)
	117 (83)	24 (17)	141 (100)
Interpersonal Losses	28 (23.9)	6 (25)	34 (24.1)
Death of a Parent	11 (9.4)	3 (12.5)	14 (9.9)
Parental Divorce	12 (10.3)	4 (16.7)	16 (11.3)
Left Home Before the Age of 18	7 (6.0)	0 (0.0)	7 (5.0)
Family Maladjustment	83 (70.9)	19 (82.6)	102 (72.9)
Psychiatric Disorder	71 (60.7)	18 (78.3)	89 (63.6)
Substance Use Disorders	50 (42.7)	8 (34.8)	58 (41.4)
Criminal Behavior	11 (9.4)	3 (13.0)	14 (10.0)
Suicide Attempts	13 (11.1)	0 (0.0)	13 (9.4)
Family Violence	35 (30.2)	8 (33.3)	43 (30.7)
Maltreatment	93 (79.5)	22 (91.7)	115 (81.6)
Neglect	62 (53.0)	16 (66.7)	78 (55.3)
Physical Abuse	76 (65.0)	18 (75.0)	94 (66.7)
Sexual Abuse	40 (34.2)	3 (13)	43 (30.7)
Life-Threatening Illness	14 (12.1)	2 (8.7)	16 (11.5)
Number of ACEs			
0	12 (10.3)	0 (0.0)	12 (8.5)
1	14 (12.0)	3 (12.5)	17 (12.1)
2	15 (12.8)	5 (20.8)	20 (14.2)
3	16 (13.7)	4 (16.7)	20 (14.2)
4	21 (17.9)	6 (25.0)	27 (19.1)
5+	39 (33.3)	6 (25.0)	45 (31.9)
At least one ACE	105 (89.7)	24 (100.0)	129 (91.5)

Table 3 displays the distribution of psychiatric disorders among adults who underwent bariatric surgery. Within this sample, 117 individuals met the criteria for at least one current psychiatric disorder (83%). The most frequent current psychiatric disorders were depressive disorder (54.6%) and anxiety disorders (54.6%). When examining lifetime psychiatric disorders, 130 individuals met the criteria for at least one disorder (92,2%). Lifetime depressive disorder was the most frequent condition (81.6%).

**Table 3:** Frequency of psychiatric disorders among adults who underwent bariatric surgery

Psychiatric Disorder	Female*n* (%)	Male *n* (%)	Total *n* (%)
	117 (83)	24 (17)	141 (100)
Current			
Bipolar Disorder	43 (36.8)	11 (45.8)	54 (38.3)
Depressive Disorder	63 (53.8)	14 (58.3)	77 (54.6)
Substance use Disorder	18 (15.4)	6 (25)	24 (17)
Anxiety Disorder	69 (59)	8 (33.3)	77 (54.6)
Eating Disorder	45 (38.5)	9 (37.5)	54 (38.3)
Any Psychiatric Disorder	97 (82.9)	20 (83.3)	117 (83)
Lifetime			
Bipolar Disorder	60 (51.3)	15 (62.5)	75 (53.2)
Depressive Disorder	96 (82.1)	19 (79.2)	115 (81.6)
Substance use Disorder	21 (17.9)	12 (50)	33 (23.4)
Anxiety Disorder	76 (65)	10 (41.7)	86 (61)
Eating Disorder	65 (55.6)	13 (54.2)	78 (55.3)
Any Psychiatric Disorder	108 (92.3)	22 (91.7)	130 (92.2)

Table 4 displays results of the association between ACE and psychiatric disorders. Based on logistic regression analysis, family maladjustment was the ACE with a significant association with bipolar disorders (current OR = 3.0 and lifetime OR = 3.5), substance use disorders (current OR = 4.3 and lifetime OR = 5.3), and anxiety disorders (current OR = 2.9 and lifetime OR = 2.3). Interpersonal losses were also associated with the presence of current (OR = 2.6) and lifetime (OR = 3.0) bipolar disorders. Childhood maltreatment was associated with any current (OR = 6.5) and lifetime (OR = 4.1) Substance Disorder. None of the ACE categories showed a significant correlation with eating disorders or the presence of current or lifetime psychiatric disorders. It was not possible to determine whether life-threatening illness predicted the presence of any psychiatric disorder, as all individuals exposed to this adversity were also diagnosed with at least one psychiatric conditio.

**Table 4:** Logistic regression models of the associations between ACE category and the presence of current and lifetime psychiatric disorders, controlling variables sex, age, GAF, BMI, and time post-surgery

	Interpersonal losses OR [CI(95%)]	Family Maladjustment OR [CI(95%)]	Maltreatment OR [CI(95%)]	Life-threatening illness OR [CI(95%)]
Current Psychiatric Disorder				
Bipolar Disorder	2.6 [1.1; 6.3]*	3.0 [1.19; 8.0]*	2.2 [0.8; 6.8]	1.2 [0.4; 3.9]
Depressive Disorder	1.4 [0.6; 3.5]	1.3 [0.57; 2.3]	1.7 [0.6; 4.6]	3.9 [1.2; 14.4]*
Substance use Disorder	0.1 [0.3; 2.8]	4.3 [1.1; 28.5]*	6.5 [1.9; 121.6]*	0.9 [0.1; 4.0]
Anxiety Disorder	1.1 [0.5; 2.5]	2.9 [1.30; 6.63]**	1.4 [0.5; 3.4]	1.3 [0.4; 4.1]
Eating Disorder	0.6 [0.2; 1.4]	1.0 [0.5; 2.4]	1.2 [0.5; 3.3]	0.9 [0.3; 2.8]
Any Psychiatric Disorder	1.2 [0.4; 4.3]	2.2 [0.8; 5.9]	2.1 [0.7; 6.3]	5.2 [0.9; 99.5]
Lifetime Psychiatric Disorder				
Bipolar Disorder	3.0 [1.3; 7.5]**	3.5 [1.5; 8.4]**	1.5 [0.6; 3.8]	1.0 [0.3; 3.1]
Depressive Disorder	1.6 [0.5; 5.5]	2.5 [0.1; 6.9]	1.0 [0.3; 2.3]	6.1 [1.1; 117.2]*
Substance use Disorder	1.7 [0.6; 4.5]	5.3 [1.5;28.4]**	4.1 [1.0; 28.1]*	0.9 [0.2; 3.6]
Anxiety Disorder	0.9 [0.4; 2.0]	2.3 [1.0; 5.1]*	1.3 [0.52; 3.2]	3.9 [1.1; 17.9]*
Eating Disorder	0.7 [0.3; 1.6]	0.7 [0.3; 1.4]	0.8 [0.34; 2.0]	1.2 [0.4; 3.8]
Any Psychiatric Disorder	1.8 [0.4; 13.0]	1.6 [0.9; 5.9]	0.4 [0.0; 2.1]	-

*p < 0.05, **p < 0.01

The number of ACEs showed no association with lifetime psychiatric disorders (*p* = 0.1). However, there was an association between the cumulative number of ACEs and current psychiatric disorders (*p* = 0.04). For each additional ACE, the odds of current psychiatric disorders were 7% greater (OR = 1.1, 95% CI: 1.0; 1.1).

## Discussion

This study assessed the frequency of ACEs among individuals undergoing MBS and explored the association between distinct ACE categories and psychiatric outcomes. A notably high proportion (> 90%) of individuals reported experiencing at least one ACE, with childhood maltreatment being the most frequently reported category. Family maladjustment correlated with a higher number of psychiatric disorders, including bipolar disorder, substance use disorder, and anxiety disorder, in both current and lifetime periods. The findings suggested that ACEs related to the family environment have a prominent association with the mental health of MBS patients. Additionally, a greater number of ACEs were associated with greater odds of current psychiatric disorders. These results emphasized the importance of integrating ACE screening into obesity management programs to enhance specialized psychological support. In accordance with the TIC framework, targeted interventions were considered beneficial for improving psychosocial outcomes and supporting weight management in patients with high ACE exposure [20].

The estimated prevalence of ACEs among patients with severe obesity ranges between 32% and 70% in a recent systematic review [38]. However, the frequency in our study was significantly higher. Several factors may explain this discrepancy. First, most research originates in high-income countries (United States/Western Europe), where lower ACE prevalence may relate to different socioeconomic conditions [4, 39]. Second, the high frequency in this study may be partially attributed to the post-operative timing of the assessments. Most studies have investigated ACEs in patients with severe obesity before surgery [37]. This pre-surgical period is a critical distinction, as many candidates for MBS may underreport stressful childhood experiences due to concerns that such disclosures could compromise their eligibility for the procedure. Peterhänsel et al. [40] demonstrated lower maltreatment reporting in patients on MBS waiting lists compared to those not seeking surgery. Conversely, the frequency observed in the present study (91.5%) is consistent with elevated rates reported in other post-MBS samples, such as the 96.8% prevalence identified by Fink et al. [41]. Therefore, the timing of data collection, pre- versus post-surgery, is a critical methodological factor in explaining these findings.

While childhood maltreatment was the most frequent ACE, family maladjustment was the category most associated with psychiatric disorders, including bipolar disorder, substance use disorder, and anxiety disorder, in both current and lifetime diagnoses. Reported maladjustments were commonly a history of a family member with psychiatric disorder, substance use, or family violence. Unlike isolated traumatic events, family maladjustment often involves chronic exposure to stressful, uncontrollable situations without supportive caregivers, triggering toxic stress responses [42, 43]. This environment may profoundly impair emotional regulation and impulse control, which are core features in patients with severe obesity [44]. Thus, chronic family maladjustment might represent not only a developmental risk factor, but also a sustained source of psychosocial stress throughout life and may be associated with persistence or exacerbation of psychiatric comorbidities.

Comparison with existing literature is challenging due to methodological heterogeneity. To date, only one study has assessed the psychopathology of patients with severe obesity using validated semi-structured interviews like the SCID [45], but it did not examine the same categories of maltreatment and neglect. In line with Wildes et al. [45], this study also found that individuals with a history of sexual abuse had increased odds of developing mood and anxiety disorders, whereas physical abuse was associated with higher rates of substance use disorders. Nevertheless, the specific role of different ACE categories on the outcome of patients with severe obesity remains uncertain. Existing studies have yielded sparse and inconsistent findings [46, 47]. Expanding this line of inquiry could provide valuable insights into targeted interventions for this population, ultimately improving their psychological well-being.

Chronic childhood stress affects the functionality of the amygdala and the hypothalamic-pituitary-adrenal (HPA) axis, leading to impaired emotional regulation and increased psychopathology [48, 49]. This exposure also elevates inflammatory markers, which may mediate the link between obesity and mental disorders [8, 42]. Consequently, a dose-response relationship was observed, in which the number of ACEs was correlated with psychiatric risk, consistent with findings reported in other clinical obesity samples [45, 47, 50, 51]. It is therefore crucial to assess not only the presence of ACEs but also their cumulative severity.

Our findings have significant clinical implications for the care of patients with severe obesity undergoing MBS. The high frequency of ACEs reinforces the need for routine screening for childhood adversity as a standard component of the preoperative psychosocial evaluation. However, screening alone is insufficient. Bariatric programs should adopt a systemic TIC framework, training multidisciplinary staff to recognize the pervasive impact of trauma on the mental health of bariatric patients [20]. This approach should include practical modifications to the care continuum, such as ensuring physical privacy in perioperative spaces, minimizing sensory triggers (e.g., ambient noise and light), and exercising heightened sensitivity during physical examinations or anesthesia induction to prevent re-traumatization [20]. For patients with a history of family maladjustment or mistreatment, which were strongly associated with psychiatric disorders in the current sample, offering evidence-based psychological interventions was considered paramount. Previous literature, including work by Sarwer et al., suggested that the presence of psychiatric disorders did not necessarily preclude successful surgical outcomes, but rather emphasized the necessity of ongoing psychosocial support [20]. Future research was suggested to move beyond association and evaluate the effectiveness of these interventions for patients who were exposed to childhood trauma and exhibited psychiatric symptoms. Furthermore, intervention-based studies could test whether pre-surgical trauma-focused therapies were associated with improved surgical readiness and postoperative adaptation, potentially providing a pathway to more personalized patient care.

This study has limitations that should be acknowledged when interpreting results. First, there is a potential for recall bias, as childhood experiences were retrospectively reported in adulthood [52]. Memory distortions, influenced by time and the current psychological state, may affect the accuracy of these reports. Second, patients may feel uncomfortable when disclosing distressing childhood experiences to a clinician during the interview, which could lead to underreporting. However, this bias is less likely to occur after surgery, when patients are less reluctant to respond to queries regarding a sensitive past. Third, the causal relationship between early exposure to ACE and later obesity and mental disorders cannot be established with this retrospective study with cross-sectional data collection. Fourth, the specific characteristics of the sample, including the small number of participants in certain clinical subgroups and the high prevalence of at least one ACE, may have limited the statistical precision of some logistic regression models, resulting in wide confidence intervals for certain odds ratios. Consequently, these findings should be interpreted with caution. Furthermore, the statistical analyses evaluated each ACE domain in separate regression models without adjusting for the co-occurrence of other adversity categories. Because childhood adversities frequently cluster and overlap, the significant findings should not be interpreted as independent effects of a single ACE domain on psychiatric outcomes. In the future, there is a need for additional large-scale and long-term studies using validated assessment tools for ACEs to evaluate their association with weight and psychosocial outcomes after MBS.

## Conclusion

In conclusion, this study revealed a remarkably high frequency of ACEs in patients with severe obesity seeking MBS, with over 90% reporting at least one event. These findings serve as a wake-up call to tailor interventions and programs to address these widespread adversities. Family maladjustment, more so than other categories of adversity, was strongly associated with bipolar disorder, substance use disorders, and anxiety disorders. Furthermore, a dose-response relationship between the number of ACEs and increased psychiatric risk was confirmed. These results have direct implications for clinical practice, suggesting that screening in bariatric settings should identify the presence and nature of adversities. The history of family dysfunction may signal the need for more intensive, trauma-informed psychological support. Future prospective research should elucidate the mechanisms by which these specific ACE profiles impact long-term weight and mental health trajectories post-MBS.

## Data Availability

The datasets generated and analyzed during the current study are not publicly available due to confidentiality and ethical restrictions but are available from the corresponding author upon reasonable request.
